# Utility of Baseline Transcriptomic Analysis of Rheumatoid Arthritis Synovium as an Indicator for Long-Term Clinical Outcomes

**DOI:** 10.3389/fmed.2022.823244

**Published:** 2022-05-03

**Authors:** Vidyanand Anaparti, Dana Wiens, Liam J. O'Neil, Erika Hubbard, Robert Robl, Irene Smolik, Carol Hitchon, Peter E. Lipsky, Hani El-Gabalawy

**Affiliations:** ^1^Manitoba Center of Proteomics and Systems Biology, Winnipeg, MB, Canada; ^2^Department of Internal Medicine, University of Manitoba, Winnipeg, MB, Canada; ^3^Ampel BioSolutions LLC, Charlottesville, VA, United States

**Keywords:** synovium, matrix metalloproteinase, microarray, long-term clinical outcomes, transcriptome, fineneedle biopsy (FNB)

## Abstract

**Objective:**

Rheumatoid arthritis is a chronic inflammatory autoimmune disease that can lead to synovial damage, persistent joint pain, and functional disability. Our objective was to evaluate baseline synovial transcriptome from early inflammatory arthritis patients (EIA) and identify pretreatment biomarkers that could potentially provide insights into long-term functional outcomes of rheumatoid arthritis (RA).

**Methods:**

Synovial biopsies from clinically inflamed knee joints were procured from either 17 EIA patients before initiation of disease modifying anti-rheumatic drug (DMARD) therapy (DMARD-naïve EIA) using the minimally invasive closed needle biopsy technique or advanced RA patients undergoing arthroplasty. Affymetrix Human Genome U133 Plus 2 microarray platform was used to profile the synovial transcriptome. The cohort was followed clinically for a median of 12.3 years, and patient data was collected at each visit. Short-term and long-term clinical outcomes were determined by assessing RA-associated clinical parameters Statistical adjustments were made to account for asynchronous clinical visits and duration of follow up.

**Results:**

Based on the transcriptomic analysis, we identified 5 differentially expressed genes (DEGs), including matrix metalloproteinase (MMP)-1 (fibroblast collagenase) and MMP-3 (stromelysin-1) in DMARD-naïve EIA patients, relative to advanced RA patients (*q* < 0.05). Dichotomous expression of MMP-1 and MMP-3 mRNA and protein was confirmed by qPCR and immunohistochemistry respectively, based on which DMARD-naïve EIA subjects were classified as MMP-high or MMP-low. Hierarchical clustering of transcriptomic data identified 947 DEGs between MMP-high and MMP-low cohorts. Co-expression and IPA analysis of DEGs in the MMP-high cohort showed an enrichment of genes that participated in metabolic or biochemical functions and intracellular immune signaling were regulated through NF-κB and β-catenin complexes and correlated with markers of systemic inflammation. Analysis of short-term clinical outcomes in MMP-high cohort showed a significant reduction in the DAS-CRP scores relative to baseline (P <0.001), whereas area under the curve analyses of modified HAQ (mHAQ) scores correlated negatively with baseline MMP-1 (*R* = −0.59, *P* = 0.03). Further, longitudinal mHAQ scores, number of swollen joints, number of DMARDs and median follow-up duration appeared to be higher in MMP-low cohort.

**Conclusion:**

Overall, our results indicate that the gene expression profiling of synovial biopsies obtained at the DMARD-naive stage in patients with EIA categorizes them into subsets with varying degrees of inflammation and can predict the future of long-term clinical outcome.

## Introduction

Early recognition and treatment initiation in rheumatoid arthritis (RA) patients using appropriately targeted disease modifying anti-rheumatic drugs (DMARD) can improve response to therapy, prevent structural damage and facilitate a long-term disease remission (1–5). A personalized approach to treatment is only possible when patients can be stratified at the time of their diagnosis and treated based on objective biomarkers that can predict response to specific DMARDs.

RA is a chronic autoimmune condition characterized by synovial cell hyperplasia, joint deformity, bone erosion, and persistent pain, leading to progressive functional disability. The disease evolves as a continuum with early synovitis being the primary clinical manifestation, which in turn is heterogenous in clinical presentation, histopathology, response to therapy and treatment outcomes (6). In fact, studies undertaken to characterize the cellular composition and molecular mechanisms underlying synovial heterogeneity have identified distinct synovial pathotypes that can refine clinical classification of RA and are associated with clinical variables such as disease duration, systemic inflammation, and radiographic progression. Moreover, patient-stratification based on baseline molecular signatures in the synovium of early RA patients was strongly associated with long-term clinical outcomes and predicted future treatment responsiveness to DMARDs, including biologics (1, 2, 7, 8).

As a means to begin to sub-set patients with early inflammatory arthritis (EIA), we applied a microarray-based strategy to evaluate the synovial transcriptome in fine-needle tissue biopsy samples from DMARD-naïve EIA patients relative to those with established RA. Furthermore, we correlated these molecular signatures with clinical outcomes collected from these individuals periodically during a 15-year longitudinal follow-up post-DMARD intervention.

## Materials and Methods

### Study Design

Samples used in this study were obtained from participants recruited into our Manitoba Early Arthritis cohort between 2000 and 2005. The study was approved by the local research ethics boards, and participants provided written informed consent. All patients were enrolled before initiation of a first DMARD. After the biopsy, patients were treated according to current guidelines for early RA. Clinical data was acquired and captured in a custom database. These individuals were followed longitudinally every 3 months for the first year, and then every 6 months thereafter or as indicated for usual care. Visit schedules varied over the course of follow-up and the clinical outcomes were recorded at each visit. No attempt was made to guide their DMARD therapy, which was solely based on clinical indications targeting clinical remission. Baseline synovial biopsies from DMARD-naïve EIA patients were obtained using a minimally invasive closed needle biopsy technique (Parker-Pearson method). All the biopsies were performed on clinically inflamed knee joints. Samples were obtained from multiple areas in each biopsied joint, and all samples were adequate for transcriptomic and immunohistopathological analysis. Two individuals had bilateral biopsies of their affected knees. As controls, we used synovial tissues from advanced RA (*n* = 6) patients that were collected from anonymous donors during joint replacement surgery. RA diagnosis was made based on fulfilling the 2010 ACR/EULAR classification criteria, as determined by a rheumatologist (HEG/CH).

### Sample Collection, Storage, and Serology

Venous blood was collected into SST^TM^ serum separation tubes (BD Biosciences) and processed as per the manufacturer's instructions. Screening for C-reactive protein (CRP), erythrocyte sedimentation rate (ESR), anti-cyclic citrullinated protein (anti-CCP) and rheumatoid factor (RF) was performed at a clinical and/or research laboratory at a single tertiary care hospital (Health Sciences Centre, Winnipeg, Manitoba, Canada).

### Assessment of Clinical Parameters

Analysis of clinical outcomes were undertaken using all the available clinical data for each study participant. Because of the asynchronous nature of clinical visits and duration of follow up, area under the curve (AUC) of modified health assessment questionnaire (mHAQ) scores normalized to duration of follow up was used to estimate the burden of specific disease manifestations such as functional disability.

### Immunohistology

H&E (hematoxylin & eosin) staining was carried out on paraffin-embedded tissues. Total cell counts were determined through light microscopy image analysis. Immunohistological analysis and quantification of the same synovial samples was undertaken of OCT-embedded tissue blocks for differentially expressed genes (DEGs) identified in the transcriptomic analysis.

### RNA Isolation, and Microarray

Tissue homogenization, and total RNA isolation (RNeasy RNA isolation kit, Qiagen Inc) was carried out on fresh synovial biopsy samples as per manufacturer's instructions. To minimize variability, we collected at least two individual samples from different locations of each joint being used for this purpose. In the case of synovial tissues obtained from patients with late-stage RA (*n* = 6) at the time of joint arthroplasty, representative samples from each synovial tissue were used to generate total RNA, which was then processed in an identical manner to the needle biopsy samples. Ten microgram of high-quality RNA (28/18S ratio >1) was extracted from these synovial biopsy samples. RNA quality was determined on Agilent Bioanalyzer using the Agilent RNA 6000 Nano kit and quantified on a Nanodrop ND-1000 spectrophotometer. Total RNA with a A260/280 > 2.0 and an RNA integrity number (RIN) > 8.0 was used for assessing synovial transcriptome. Microarray analysis was performed by Dr. Chao Lu (Centre for Applied Genomics, Hospital for Sick Children, Toronto). Briefly, total RNA was converted to complementary RNA (cRNA) and hybridized to Affymetrix HU133plus2 chips that had 54,675 probe sets corresponding to the entire human genome. Hybridized chips were scanned using an Affymetrix Genechip Scanner 3000.

### Data Analysis and Statistics

Data from the microarray chips was normalized and analyzed using the MAS 5.0 algorithm, then imported into ArrayAssist software (Stratagene) and analyzed using Significance Analysis of Microarray (SAM) analysis (Stanford, California) (9). Mann-Whitney *U*-test, Chi-square test, Pearson correlation and Spearman rank correlation analyses were used as and when required. Graphpad Prism (v9.1) was used for graphical representation of the results. R packages or Ingenuity Pathway Analysis were used to perform functional network analysis as explained below.

### Weighted Gene Co-expression Network Analysis and Multi-Scale Embedded Gene Co-expression Network Analysis

WGCNA algorithm was used to construct co-expressed gene network modules that were assessed further for their functional significance (10). Briefly, raw microarray data files underwent background correction and GCRMA normalization resulting in log_2_ intensity values compiled into an expression set object (e-set). The e-set was then restricted to the top 5,000 probes with the highest variance among the DMARD-naïve EIA samples. A scale-free topology matrix (TOM) was calculated to encode the network strength between probes with a soft thresholding power of 30. TOM distances were used to cluster probes into WGCNA modules. Resulting co-expression networks were trimmed using dynamic tree cutting and the deepSplit function in R. Partitioning around medoids (PAM) was also utilized to assign outliers to the nearest cluster. The resulting network was formed with a minimum module size of 100, cut height of 1, and merge height of 0.2. Modules were given random color assignments and expression profiles summarized by a module eigengene (ME). Final membership of probes representing the same gene were decided based on strongest within-module correlation to the ME value. For each module, ME values were correlated by Pearson correlation to the clinical data including cohort (MMP-high group = 1, MMP-low group = 0), ESR, CRP, age, sex, swollen joints, disease duration, tender joints, and total affected joints. Significance was determined using an adjusted *p*-value ≤ 0.2. Additionally, MEGENA was applied to the dataset as an independent method of identifying co-expression networks. MEGENA is a multi-scale co-expression gene clustering algorithm, which was used to create additional gene expression networks by applying it on the normalized and filtered e-set as described for WGCNA. Multi-scale clustering structures were identified using planar filtered networks and resultant gene co-expression modules were also correlated to clinical metadata as previously described for WGCNA. The top 40 co-expression modules with significant correlations to the clinical trait of interest, cohort (MMP-high vs. MMP-low) were reported (11).

### Functional Annotation of Gene Expression Networks

Co-expression modules were annotated according to the top overlapping functional category with the most significant *p*-value and a minimum of four overlapping genes. In the absence of significant overlaps, “unknown” was the assigned annotation. Functional enrichment within the gene co-expression modules and relative significance with clinical outcomes was calculated using gene ontology (GO), transcriptomic signatures derive from published literature and functional aggregation tools, namely Immune/Inflammation-Scope (I-Scope), Tissue-Scope (T-Scope) and Biologically Informed Gene Clustering (BIG-C) (12–16). I-Scope categorizes gene transcripts into one of a possible 28 hematopoietic cell categories based on matching transcripts known to mark various types of immune/inflammatory cells. T-Scope is an additional aggregation tool to characterize cell types found in specific tissues. BIG-C classifies genes into 53 different groups based on their most probable biological function and/or cellular or subcellular localization. Odds ratios and overlap *p*-values were calculated using Fisher's Exact test in R using the fisher test function. Statistical significance was obtained using an adjusted *p*-value ≤ 0.2.

## Results

### Study Population

In total, 15 DMARD-naïve EIA patients were enrolled in this longitudinal study and underwent baseline synovial biopsy of an affected knee joint using the Parker-Pearson technique, prior to initiation of their first DMARD. Two of these study participants in whom both knees were affected underwent bilateral synovial biopsy. Table 1 and Supplementary Table S1 summarizes the clinical characteristics of EIA subjects at baseline and the list of DMARDs prescribed to them throughout the entire duration of longitudinal follow-up. Of this study population, 12/15 (80%) were female, median age was 49 years, and the median disease duration was 6 months. While 8/12 (63%) EIA subjects were seropositive for anti-CCP (median = 64, range = 12 to >201), 10/15 (67%) EIA patients were seropositive for RF (median = 385 IU, range 86–1,440), 11/15 (73%) had elevated a CRP and/or ESR. Median (range) swollen and tender joint counts (66/68 joints assessed) were 6.5 (2–28) and 8 (2–35), respectively. The calculated median DAS-CRP score was 5.1 for the group, indicating that most of the study subjects had active inflammatory arthritis.

**Table 1:** Baseline characteristics of DMARD-naïve EIA patients: All values are either reported as mean (standard deviation, SD), *n* (%) or median (range), as needed.

	**DMARD-naïve EIA**
	**(*n* = 15)**
Age, years, mean (SD)	49 (14.8)
Female, n (%)	12 (80%)
CRP, mg/L, mean (SD)	35.4 (29.1)
DAS-CRP, mean (SD)	5.1 (1.2)
RF titer, IU/mL, mean (SD)	350.9 (452.6)
ESR, mean (SD)	47.4 (23.2)
Swollen Joint Count^$^, median (range)	6.5 (2–28)
Tender Joint Count^$^, median (range)	8 (2-35)
Total Joint Count^$^, median (range)	13 (2-35)
Disease duration, months, mean (SD)	16.4 (24)
Follow-up time, months, mean (SD)	111.7 (70.6)
mHAQ, median (range)	0.25 (0–0.88)

$ *Based on a 68 joint count*.

*RA, Rheumatoid Arthritis; RF, rheumatoid factor; DAS, disease activity score; CRP, C-reactive protein; ESR, erythrocyte sedimentation rate; mHAQ, modified health assessment questionnaire*.

### Transcriptomic Analysis of Synovial Biopsies Reveals Unique MMP Signature

Affymetrix microarray was used to characterize the transcriptome in each synovial tissue. The datasets generated were normalized using the Robust Multichip Average (RMA) technique and analyzed using Stanford Analysis of Microarray (SAM) software to identify transcripts which exhibited significant variability within the transcriptomes.

**Figure 1:**
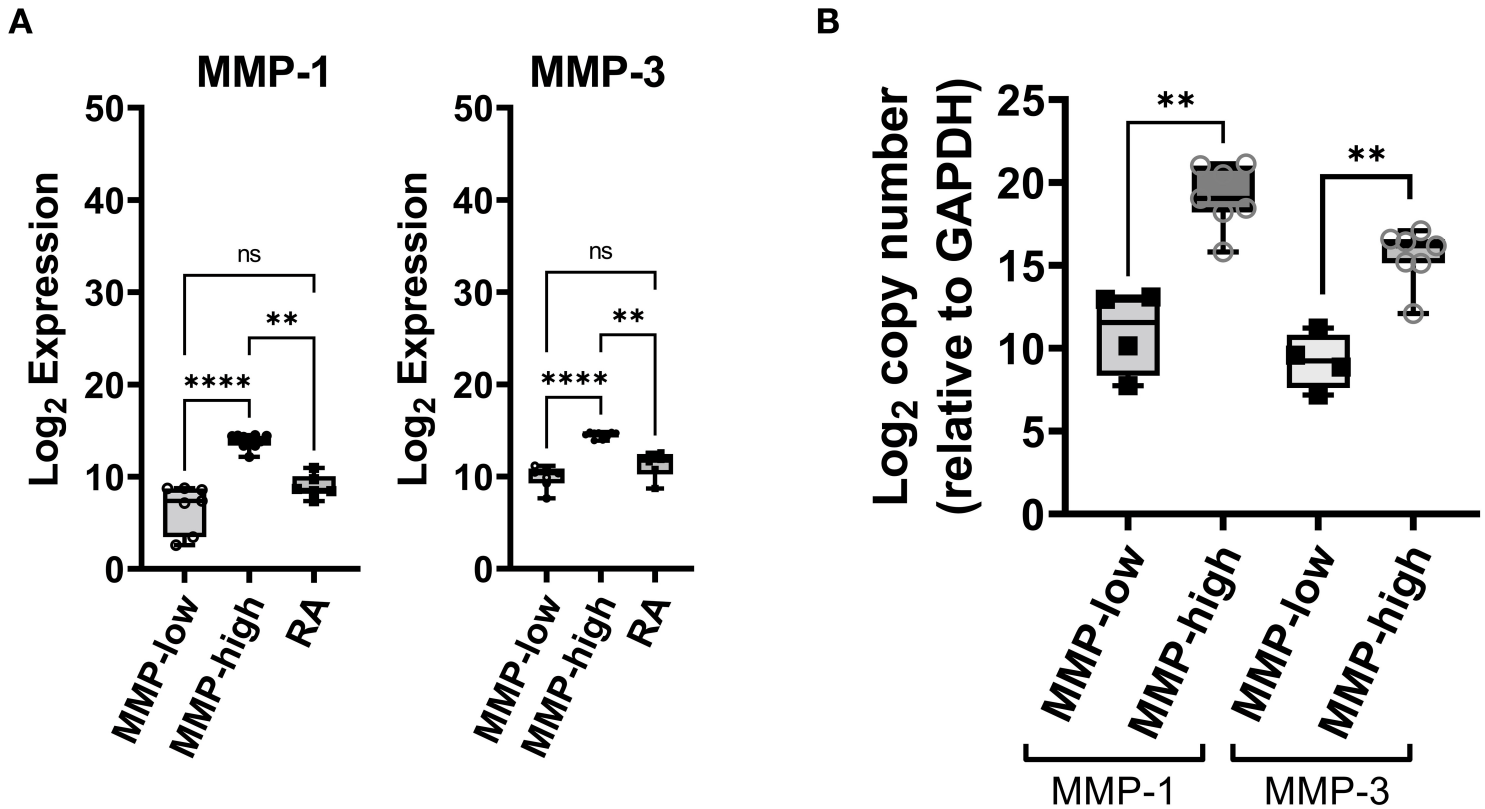
Analysis of synovial gene expression in DMARD-naïve EIA patients—**(A)** Box-whiskers plot showing the abundance MMP-1 and MMP-3 transcripts between DMARD-naive (MMP-low, MMP-high) EIA patients and advanced RA patients. Transcript abundance was determined by microarray chips. Data is presented as log_2_ expression values and analyzed using Kruskal-Wallis test with a Dunn's *post-hoc* test. ****P < 0.0001, **P < 0.01, ns, non-significant. **(B)** Box-whiskers plot showing mRNA expression of MMP-1 and MMP-3 between MMP-high and MMP-low groups. Data was generated using qPCR, presented as log_2_ copy number and analyzed by Mann-Whitney test. **P < 0.01.

After adjustment for a false discovery rate of <10%, a total of 20 mRNA transcripts (corresponding to 17 unique genes) were either significantly up- or down-regulated in the synovial biopsy samples from DMARD-naïve EIA patients compared to samples from advanced RA patients (Supplementary Table S2). Of these, MMP-1, MMP-3, CD82, VCAM1 and CHES1 (fold change = 76.87, 19.41, 1.73, 2.65, and 5.83 respectively; Supplementary Table S2) were the most significantly up-regulated genes. Because MMP-1 and MMP-3 are produced abundantly by the synovial lining layer and are known to play a key role in the progressive joint damage that occurs in RA (17, 18), we focused on these two molecules as potential biomarkers for classifying the early inflamed synovium (Figure 1). We noted a dichotomous distribution in the transcript levels of both MMP-1 and MMP-3 in the 17 EIA synovial tissue samples (including two individuals who had bilateral synovial biopsy samples). As such, 10/17 synovial tissues (60%) exhibited high transcript levels of both MMP-1 and MMP-3, and 7/17 (40%) exhibited low transcript levels, the latter being comparable to the levels detected in the advanced RA samples (Figure 1A). We confirmed the MMP-1 and MMP-3 expression levels using qPCR (Figure 1B) and showed that there was a very strong correlation between MMP-1 and MMP-3 mRNA levels in the DMARD-naïve EIA patients (*r* = 0.8897, *P* = 0.001, Supplementary Figure S2). Moreover, in analyzing the microarray datasets, we showed that this dichotomous distribution was unique to MMP-1 and MMP-3 as it was not demonstrable with any other MMP or TIMP transcripts, except for MMP-13, where similar trends were observed (Supplementary Figure S1; Supplementary Table S3). Importantly, in the two individuals who had bilateral synovial samples obtained from both their affected knees joints, there was concordance in the MMP-1 and MMP-3 transcript levels between the two knee joints of the same individual, with one patient exhibiting bilateral high levels, and the other bilateral low levels. This suggests that the MMP-1 and MMP-3 transcript levels were reflective of the individual's pathologic process and not simply related to local factors in each joint. Based on these findings, we then categorized each EIA patient as being either an MMP-high or MMP-low mRNA expressor in their inflamed synovial tissue.

### MMP-1 and MMP-3 Protein Expression in EIA Synovium, but Not in the Circulation, Is Concordant With Synovial mRNA Transcript Levels

**Figure 2:**
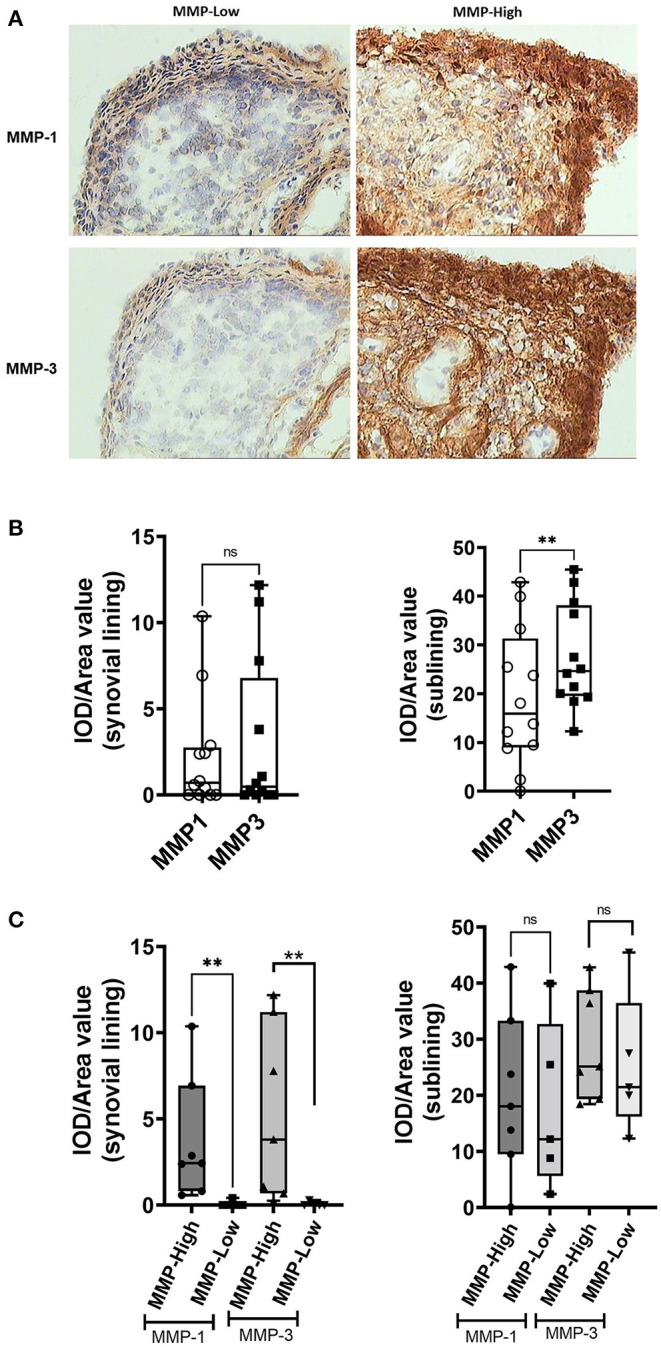
Analysis of MMP-1 and MMP-3 protein expression in the synovium of DMARD-naïve EIA patients—**(A)** Representative images showing immunohistochemical staining of MMP-1 (upper) and MMP-3 (lower) in the OCT-embedded tissue sections of MMP-low and MMP-high groups. **(B)** Box-whiskers plot showing quantification of MMP-1 and MMP-3 IHC staining in the synovial lining and sublining of DMARD-naïve EIA patients. Data was presented as IOD/area value indicating relative expression and analyzed by Mann-Whitney test. **P < 0.01, ns, non-significant **(C)** Box-whiskers plot showing quantification of MMP-1 and MMP-3 IHC staining in the synovial lining and sublining of MMP-low and MMP-high DMARD-naïve EIA patients. Data was presented as IOD/area value indicating relative expression and analyzed by Mann-Whitney test. **P < 0.01, ns, non-significant; IOD, integrated optical density.

We sought to determine whether the grouping of the EIA samples based on MMP-1/MMP-3 synovial mRNA transcript levels was reflected in the expression of the corresponding proteins, both locally in the synovial tissue using IHC (Figure 2A), and systemically in the circulation using ELISA. As shown in Figures 2B,C, there were dramatic differences in the expression of both MMP-1 and MMP-3 protein in the synovium between the MMP-high and MMP-low mRNA groups. Compared to the MMP-low group, the MMP-high group exhibited higher intensity of IHC staining for MMP-1 and MMP-3 in the synovial lining layer and in the sub-lining areas (Figure 2C). Although much of the staining appeared to be extracellular, we were able to demonstrate intense intracellular staining for these proteins in the synovial lining cells (Figure 2A). In contrast to levels observed in the synovial biopsy samples, circulating MMP-1 and MMP-3 levels did not show similar dichotomy (Supplementary Figure S3), although the levels of these two proteins were highly concordant in the serum (*r* = 0.5473, *P* = 0.0478), and correlated with the degree of inflammation as indicated by CRP levels (Supplementary Figure S3).

### Delineation of a Synovial Transcriptomic Signature Based on the MMP-1/MMP-3 Grouping

**Figure 3:**
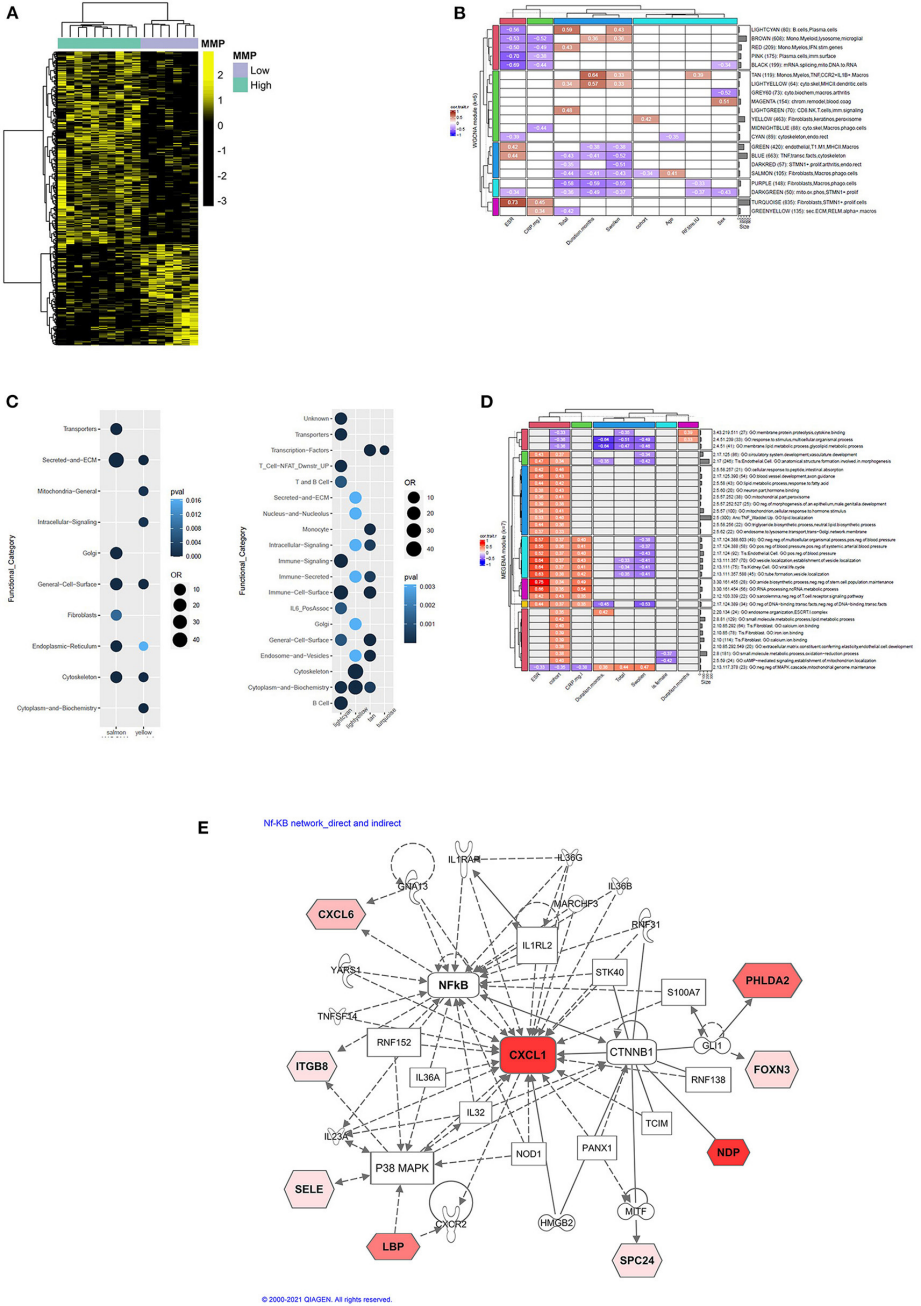
**(A)** Heatmap showing unsupervised hierarchical clustering of differentially expressed genes (DEGs) between MMP-high and MMP-low groups. Genes were clustered using Pearson correlation and complete linkage clustering algorithms. **(B)** Hierarchical clustering plot showing 21/23 WGCNA modules with significant correlations to at least one clinical trait of the 17 EIA samples. Pearson correlation coefficients are colored according to the legend and values overlaid in their respective cells. **(C)** Pearson correlation plots showing interactions between WGCNA modules and functional pathways. **(D)** Hierarchical clustering plot showing Pearson correlation between MEGENA modules and clinical variables. **(E)** Ingenuity pathway analysis (IPA) showing curated molecular interactions between DEGs in MMP-high cohort. Molecules highlighted in red (or shades of red) represent DEG that are elevated in MMP-high cohort.

We applied unsupervised hierarchical clustering algorithm to identify the spectrum of differentially expressed genes (DEGs) within the microarray dataset between MMP-high and MMP-low groups (Figure 3A). Analysis revealed the presence of two distinct clusters based on the gene expression profile. While 622 genes were found to be increased in MMP-high subjects, expression of 325 genes was high in the MMP-low group.

WGCNA and MEGENA analysis was applied to the transcriptomic data to explore gene co-expression modules and determine biological processes that drive their differential expression in DMARD-naïve EIA subjects (Figures 3B–D). WGCNA analysis yielded 23 co-expressed gene modules (each module was assigned a color), 21 of which correlated with at least one clinical outcome (Figure 3B). Of these, salmon and yellow were the only modules that showed association with the MMP-status of the cohort (*r* = −0.34 and *r* = 0.42, respectively). Therefore, we selected these modules for further analysis. As shown in Figure 3C, subsequent assessment of the yellow module for functional relevance revealed an enrichment for genes involved in metabolic functions (Mitochondria, Cytoplasm and Biochemistry) and was associated with MMP-high group. On the other hand, the salmon module was enriched for fibroblast and stromal signatures (Cytoskeleton, Secreted and ECM) and associated with the MMP-low group (Figure 3C). We also interrogated gene expression signature in the lightcyan, lightyellow, tan and turquoise modules owing to their correlation with total affected joints, swollen joints, disease duration, ESR and CRP. These modules were enriched for B cell, T cell, and IL-6 gene signatures (Figure 3C). MEGENA was applied to further dissect complex co-regulatory gene networks and explore their interaction with clinical outcomes in DMARD-naïve EIA patients (Figure 3D). A majority of these modules correlated positively with MMP-status of the patient, followed by ESR and CRP. Most importantly, parent modules were found to be enriched for inflammatory and phagocytic macrophage-associated gene signatures, including those involved in TNF signaling. We also observed an enrichment of the fibroblast transcriptomic signature in module 2.10 and related modules.

We then applied IPA bioinformatics tool to identify common transcriptional hubs that were primarily responsible for differential expression of certain genes in MMP-high patients. Analysis of curated functional networks revealed direct and indirect relationships with multiple cell-signaling molecules that were centrally connected to NF-κB, β-catenin (CTNNB1) and p38MAPK, and converge leading to increased CXCL1 expression (Figure 3E). Most of these molecules were acute-phase proteins involved in IL-17A signaling in autoimmune diseases such as psoriasis and arthritis (14.3% overlap; *P* = 0.0000266).

### High Baseline MMP1 and MMP3 MRNA Levels in the Inflamed Synovium Are Associated With Better Long-Term Outcomes

**Figure 4:**
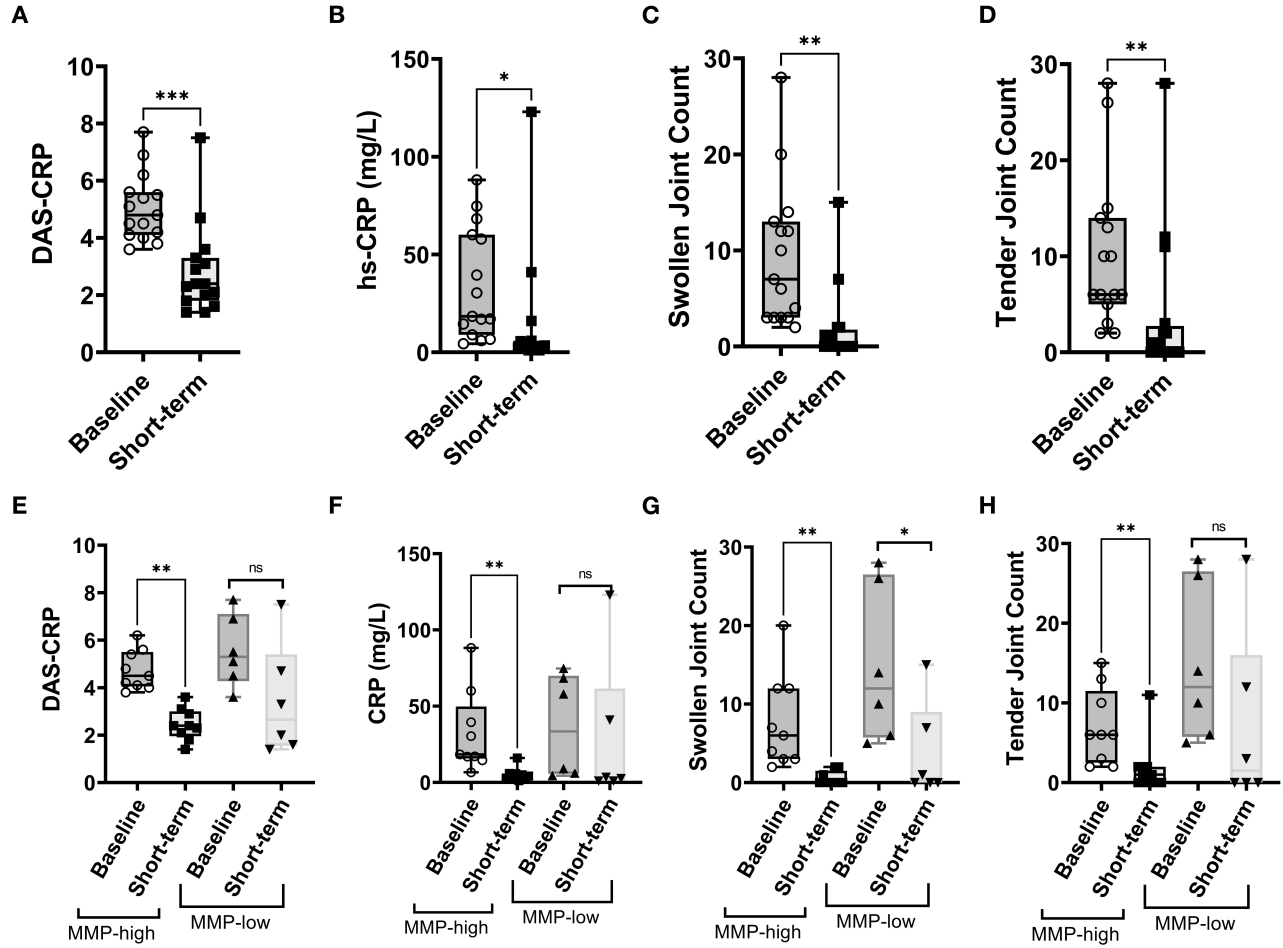
Analysis of short-term clinical outcomes in DMARD-naïve EIA patients – **(A–D)** Box-whiskers plot showing DAS-CRP, hs-CRP levels (represented as mg/L), swollen joint count and tender joint count at baseline and short-term follow-up interval. Data was analyzed by Mann-Whitney test. *** *P* < 0.001, * *P* < 0.05 **(E–H)** Box-whiskers plot showing DAS-CRP, hs-CRP levels (represented as mg/L), swollen joint count and tender joint count at baseline and short-term follow-up interval in MMP-low and MMP-high groups. Data was analyzed by Mann-Whitney test. ** *P* < 0.01, ns = non-significant.

**Figure 5:**
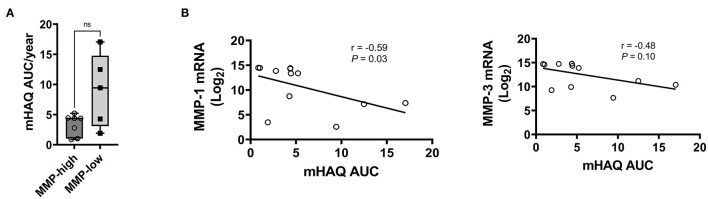
Analysis of long-term clinical outcomes in DMARD-naïve EIA patients—**(A)** Box-whiskers plot showing mHAQAUC/year after 12.5 years of follow-up between MMP-high and MMP-low groups. Data was analyzed by Mann-Whitney test; ns, non-significant **(B)** Pearson correlation plots showing the relationship between MMP-1 or MMP-3 levels at baseline and mHAQ AUC scores at 15 year follow-up.

Given the distinct baseline synovial transcriptomic signatures identified in the cohort of individuals with early, untreated inflammatory arthritis, we next sought to determine whether there were differences in the longitudinal clinical outcomes, when categorized based on MMP dichotomy. Patients enrolled in the study were followed for a median of 12.3 years (# clinical visit = 24; IQR 16). Clinical assessment (swollen and tender joints), functional scores (mHAQ) and medications were recorded at each clinical assessment. Baseline clinical features were similar between MMP-high and MMP-low groups (Supplementary Table S5). At the short-term follow-up interval of 31 months, DMARD-naïve EIA patients displayed a significant reduction in their CRP levels, DAS-CRP scores, swollen joint counts and tender joint counts relative to the levels at their baseline visit (Figures 4A–D). Interestingly, these changes were highly prominent in the MMP-high group compared to the MMP-low patients (Figures 4E–H). To determine the burden of long-term functional disability, we next assessed mHAQ scores across all longitudinal visits, normalized to follow-up duration (mHAQ AUC/year). Longitudinal mHAQ scores showed an increasing trend in MMP low patients compared to MMP high (Figure 5A; 9.5 vs. 4.4, *P* = 0.20), albeit insignificant. Similar trends were observed in swollen joint counts and number of DMARDs, a surrogate for treatment resistant disease (Table 2), based on the MMP status at arthritis onset. Importantly, baseline MMP-1, and to a lesser extent MMP3, mRNA levels showed an inverse correlation with long-term mHAQ AUC scores (Figure 5B). We also observed that the median length of follow up was longer for MMP-low patients (177 months) compared to MMP-high (74 months), though the number of annual visits were similar between the groups (MMP-high vs. MMP-low; 2.03 vs. 1.96 per year). Together, these data suggest that the MMP-high group appeared to accrue less long-term disability compared to the MMP-low group.

**Table 2:** Long-term clinical outcomes in MMP-high and MMP-low DMARD-naïve EIA patients.

**MMP status**	**Follow-up time (months)**	**No. of visits (total)**	**mean mHAQ**	**mHAQ AUC/yr**	**Mean # swollen joints**	**Mean # swollen joints AUC/yr**
**MMP-high**
ERA 133	195	29	0.32	2.78	0.8	10.76
ERA 123	60	11	0.16	1.03	1.36	10.2
ERA 135	50	8	0.39	4.44	0.75	8.13
ERA 115	183	31	0.52	4.37	1.5	0.69
ERA 95	182	30	0.08	0.86	1.39	14.97
ERA 82	74	17	0.49	5.22	7.38	79.58
ERA 144	26	5	0.29	4.38	1.5	17
**Median**	74	17	0.32	4.37	1.39	10.76
**MMP- Low**
ERA 116	198	25	0.39	4.28	1	7.62
ERA 110	178	29	1.09	12.49	2.83	27.47
ERA 3	117	34	0.29	1.9	5.52	28.43
ERA 143	177	23	0.81	9.43	3.15	29
ERA 25	74	14	1.29	17.05	5.15	69.17
**Median**	177	25	0.81	9.43	3.15	28.43

*mHAQ, modified health assessment questionnaire; AUC, area under the curve*.

## Discussion

We present the results of a broad transcriptomic analysis of baseline synovial tissue samples that were obtained using closed needle biopsy from DMARD-naïve EIA patients with inflammatory arthritis, most of whom were diagnosed with seropositive early inflammatory arthritis. We defined the transcriptomic signature that was predictive of long-term clinical outcomes in these patients who all received standard care of treatment.

The synovium is the primary target organ for the chronic immuno-inflammatory process that characterizes RA, and other chronic arthropathies (19). It is also well-established that both the systemic and synovial responses to a wide array of available DMARD/biologic therapies is heterogeneous, and notoriously difficult to predict based on clinical parameters and circulating biomarkers such as autoantibody profiles and CRP. This challenge is further complicated by the unpredictable loss of therapeutic efficacy to currently available RA drugs, necessitating empiric trials of alternative therapies in the hope of recapturing control of the disease. Because of this, there has been a longstanding interest in identifying predictive synovial biomarkers early in the disease process that could help classify the inflammatory lesions based on pathotypes which could, in turn, potentially inform difficult clinical decisions (7, 20). Much progress has been made in this area, particularly recently, where large international consortia have provided intriguing new data based on state-of-the-art analyses of the synovial biopsies (3, 7, 21). Yet, despite the availability of sizable cohorts of RA patients who have undergone synovial biopsy in research settings, a key gap is the lack of data regarding the long-term outcomes of these biopsied RA patients in routine clinical settings where individuals typically cycle through several agents, alone or in combination. The data presented in the current study are an attempt to address this gap by providing longitudinal outcome data in a cohort of RA patients who underwent baseline synovial biopsy and were then followed for up to 15 years under routine clinical care.

Overall, we showed that the transcriptional signature of the synovium of DMARD-naïve patients with active RA was heterogenous, and this heterogeneity was primarily defined by dichotomous expression of MMP-1 and MMP-3 genes, both at the gene and protein level. Characterization of molecular pathways underlying divergent synovial MMP1/MMP3 expression suggests the presence of distinct types of synovitis, one of which is regulated by NF-κB and β-catenin. Importantly, RA patients with high MMP1/MMP3 expression exhibited a significant reduction in their disease activity, and inflammation at a short-term follow-up point and improved physical function when assessed after a prolonged period of DMARD therapy. We also observed a difference in the mRNA levels of MMP-13 between MMP-high and MMP-low EIA subjects. However, these levels did not achieve statistical significance when corrected for false-discovery rate using Benjamini-Hochberg method. In contrast, short and long-term treatment response in MMP-low cohort was reminiscent of outcomes observed in treatment resistant individuals. Taken together, our data suggests a strong association between baseline MMP-status of the synovium and response to DMARD treatment, thereby underscoring the diagnostic value of synovial transcriptome at the pre-DMARD stage as a predictor of response to RA therapy. Additional studies are needed to evaluate whether baseline synovial MMP-status can be used as an indicator to determine short and long-term response to a specific RA drug or is applicable to any DMARD therapy (2, 3, 7, 22).

To determine the potential clinical utility of the baseline synovial MMP grouping, we evaluated the clinical outcomes of the cohort over an extended longitudinal timeframe. We defined relatively short-term outcomes after an average of ~2 years, and long-term outcomes after more than one decade. No attempt was made to guide the subsequent DMARD/biologic therapy these individuals received, and they were treated using standard of care. As such, we had serial visits for each member of the cohort, with documentation of joint counts and mHAQ scores. Our future work will explore the relationship between synovial gene expression and the accrual of radiographic joint damage in both the biopsied joint and non-biopsied joints of hands and feet. Since no one visit could be regarded as an endpoint, we chose to perform an area under the curve (AUC) analysis as a method to quantify longitudinal outcomes. Our study also has other important limitations, the most obvious of which is the small size of the clinical cohort. This may have introduced bias toward specific synovial pathotypes, and indeed may limit the generalizability of the findings. Also, we acknowledge the lack of information on the medications prescribed to advanced RA patients, who were anonymous donors undergoing a joint replacement surgery. Despite these shortcomings, we were able to clearly delineate two major synovial subsets based on the levels of MMP-1 and MMP-3 mRNA and protein expression, both of which paralleled each other. These two MMPs are known to play a key role in the pathogenesis and destructiveness of inflammatory arthritis (17, 23, 24). Importantly, at baseline, the two groups were clinically indistinguishable suggesting that there may be potential clinical utility to assessing their synovial expression. As such, it is important to point out that the circulating levels of the MMPs did not correlate with their synovial expression levels. Using this approach, we unexpectedly demonstrated that the group with the highest baseline synovial expression levels of MMP-1/MMP-3 appeared to accrue less functional disability over time than the group with substantially lower levels, the latter being comparable to the levels we demonstrated in synovial samples obtained from RA patients at the time of joint arthroplasty. This finding seems to be counter intuitive considering the role these molecules play in the destruction of cartilage and connective tissue in the synovial compartment (24). One potential explanation for this unexpected finding is that the individuals with a high MMP-1/MMP-3 baseline signature are more responsive to DMARD therapy. This may be analogous to observations made in the context of malignancies where highly proliferative, activated neoplasms respond better to chemotherapy than do those that are more indolent (25). However, it needs to be validated in other longitudinally followed early RA cohorts and confirm our observations.

Evidence suggests synovial phenotypes can range from a myeloid pattern to a lymphoid or a fibroid phenotype defined primarily by the cell-types infiltrating into the synovium [1, 10, 12]. Our gene expression analysis or immunohistological staining did not show any evidence of either myeloid lymphoid or pauci-immune phenotypes prior to DMARD therapy. We also observed a homogenous infiltration of macrophages (CD68), fibroblasts (CD55) and lymphocytes (CD3 and CD20) in the synovium. This may be due to a low sample size, use of a different methodology for identifying gene expression or a different patient population. The co-expression analysis identified an enrichment of metabolic, and inflammatory genes in the MMP-high subtype, most of which are involved in active intracellular signaling. Most of these pathways, including those targeted by DMARDs regulate the expression of molecules that are involved in the NF-κB pathway (26). In contrast, MMP-low samples showed a predominance of fibroblast genes that are involved in ECM and cytoskeletal reorganization. IPA curated analysis of genes enriched in MMP-high group identified NF-κB complex, and β-catenin as the two major transcriptional nodes. In patients with early inflammatory arthritis, NF-κB activation in the synovium facilitates proliferation of synovial fibroblasts, modulates tissue-specific immune responses, and perpetuates chronic inflammation by promoting secretion of pro-inflammatory mediators including MMP1 and MMP-3 in the synovium (26–29). Further investigations are needed on synovial biopsies collected longitudinally at different time intervals from patients on DMARD therapy to validate these hypotheses and shed light on how molecular processes evolve within the synovium in response to treatment in individuals stratified according to their baseline tissue MMP-status.

In conclusion, we have demonstrated an MMP-centered synovial heterogeneity in DMARD-naïve EIA patients, which could reliably predict short-term and long-term response to treatment irrespective of the DMARD being administered. If this approach is validated, it may be a valuable metric for clinicians to identify individuals who could have homogenous response to specific treatments and allow them to provide the right treatment to right patient, a step toward precision medicine in RA.

## Data Availability Statement

The original contributions presented in the study are publicly available. This data can be found here: https://www.ebi.ac.uk/arrayexpress/experiments/E-MTAB-11274.

## Ethics Statement

The Manitoba Early Arthritis Cohort study was approved by the University of Manitoba's research ethics boards, and participants provided written informed consent.

## Author Contributions

HE-G and PL conceived research concept. VA, DW, LO'N, HE-G, RR, and EH analyzed data. VA, DW, and EH prepared figures. CH recruited patients, acquired samples, and collected longitudinal clinical data. VA, DW, LO'N, EH, PL, and HE-G drafted and revised the manuscript, and all co-authors participated in editing the manuscript. All authors contributed to the article and approved the submitted version.

## Funding

Manitoba Early Arthritis Cohort study was funded by serial grants from the Health Sciences Research Foundation (Principal Investigator: CH). The study was also supported by individual grants to HE-G from the Canadian Institutes of Health Research and from AstraZeneca UK. The funder was not involved in the study design, collection, analysis, interpretation of data, the writing of this article or the decision to submit it for publication. VA received Postdoctoral fellowships from Research Manitoba and Arthritis Society of Canada. EH, RR, and PL were supported by a grant from the RILITE foundation.

## Conflict of Interest

EH, RR, and PL are employed by Ampel BioSolutions, LLC. The remaining authors declare that the research was conducted in the absence of any commercial or financial relationships that could be construed as a potential conflict of interest.

## Publisher's Note

All claims expressed in this article are solely those of the authors and do not necessarily represent those of their affiliated organizations, or those of the publisher, the editors and the reviewers. Any product that may be evaluated in this article, or claim that may be made by its manufacturer, is not guaranteed or endorsed by the publisher.
