# Supplementary material for: Utility of Baseline Transcriptomic Analysis of Rheumatoid Arthritis Synovium as an Indicator for Long-Term Clinical Outcomes
Source: Front Med (Lausanne). 2022 May 3;9:823244. doi: 10.3389/fmed.2022.823244 (PMC9110862; doi:10.3389/fmed.2022.823244)
Supplement: Supplementary file 1 [file Data_Sheet_1.docx]

**Table S1:** Table showing disease modifying anti-rheumatic drugs (DMARDs) prescribed to EIA patients at first visit, short-term follow-up, and long-term follow-up timepoints. NA = details unavailable.

| **ERA ID** | **First prescribed** | **Short term**  **follow-up** | **Long-term**  **follow-up** |
| --- | --- | --- | --- |
| 04 | etanercept | NA | NA |
| 07 | methotrexate + hydroxychloroquine | NA | NA |
| 08 | etanercept | NA | NA |
| ERA 03 | etanercept | methotrexate + etanercept | adalimumab + methotrexate |
| ERA 25 | methotrexate | methotrexate | leflunomide |
| ERA 82 | methotrexate | methotrexate + leflunomide | etanercept |
| ERA 95 | sulfasalazine | methotrexate | methotrexate + sulfasalazine |
| ERA 110 | methotrexate | methotrexate + sulfasalazine + hydroxychloroquine | adalimumab |
| ERA 115 | methotrexate + sulfasalazine | Methotrexate | methotrexate + certolizumab |
| ERA 116 | methotrexate + sulfasalazine | methotrexate + sulfasalazine | methotrexate |
| ERA123 | methotrexate | Methotrexate | methotrexate |
| ERA 133 | NA | Methotrexate | adalimumab |
| ERA 135 | methotrexate | Methotrexate | methotrexate |
| ERA 143 | NA | methotrexate + infliximab | methotrexate |
| ERA 144 | NA | NA | NA |
| ERA 154 | NA | rituximab + methotrexate + hydroxychloroquine | methotrexate + sulfasalazine + hydroxychloroquine |
| ERA 165 | NA | methotrexate + sulfasalazine + hydroxychloroquine | etanercept + methotrexate + sulfasalazine + hydroxychloroquine |

**Table S2:** Table showing differential expression of gene transcripts between ERA and RA patients. Data was analyzed by Student t test and corrected for false-discovery rate.


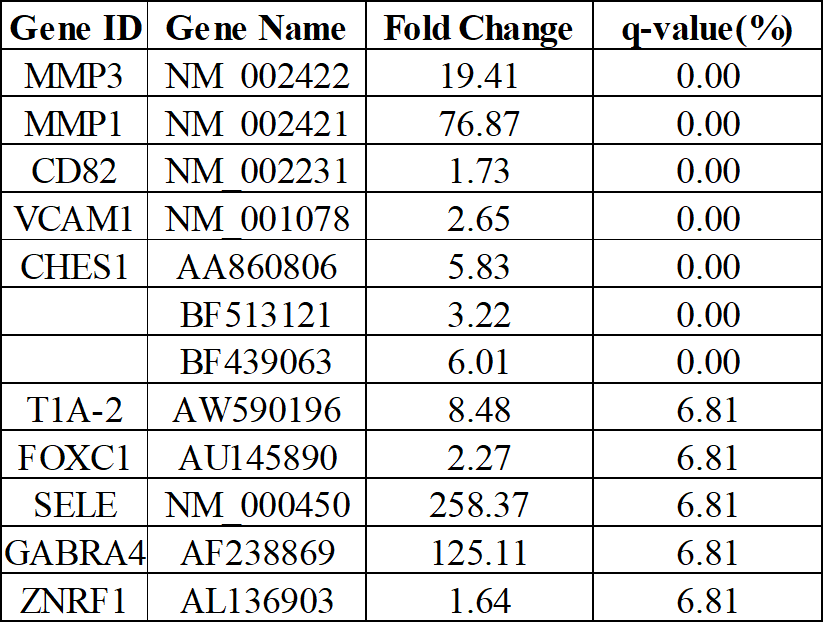


**Fig S1**: Violin plot showing the transcript abundance of various MMPs in ERA patients


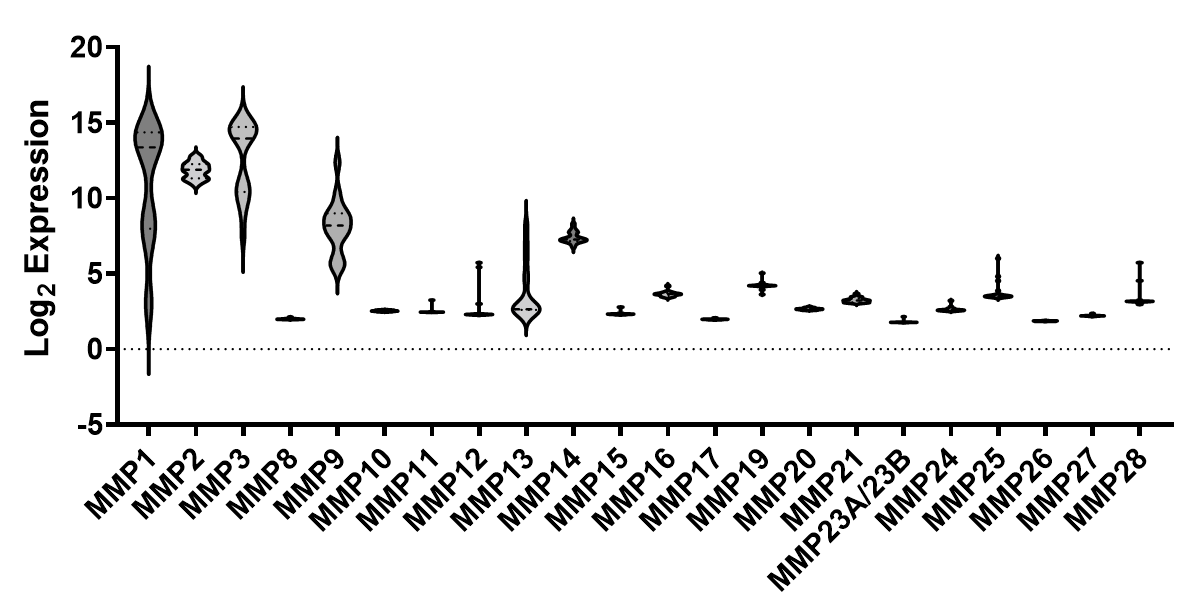


**Table S3:** Table showing the mRNA abundance of MMPs and TIMPs in MMP-high and MMP-low groups. Data is represented as log2-normalized values and was analyzed by independent samples T test.


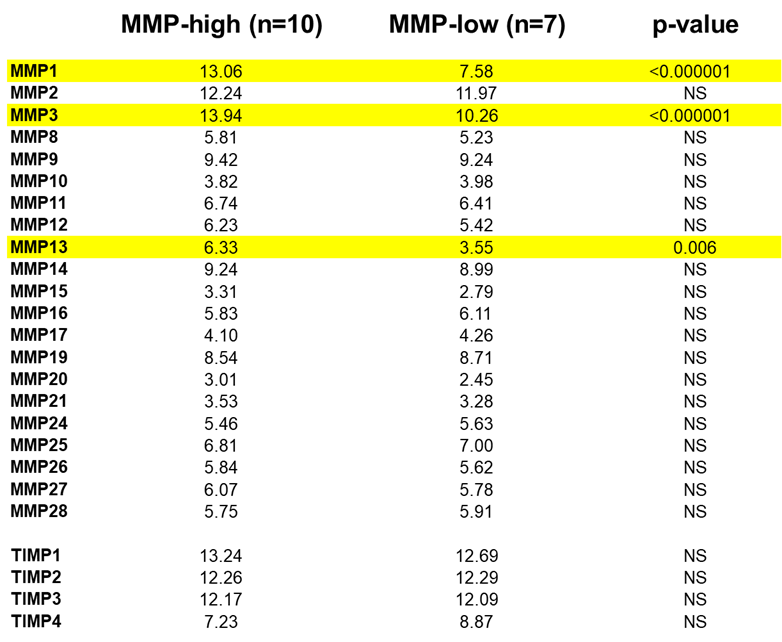


**FigS2:** Spearman rank correlation analysis between MMP-1 and MMP-3 mRNA levels in ERA patients (open circles = MMP-high; close circles = MMP-low)

**Fig S3**: (A) Box-whiskers plot showing the expression of MMP-1 and MMP-3 in the serum of MMP-high and MMP-low ERA subjects. Data was analyzed by Mann-Whitney U test; ns = non-significant. (B) Spearman rank correlation analysis between serum MMP-1 and MMP-3 levels in ERA patients (open circles = MMP-high; close circles = MMP-low) (C & D) Spearman rank correlation analysis between serum MMP levels and hs-CRP in ERA patients (open circles = MMP-high; close circles = MMP-low)


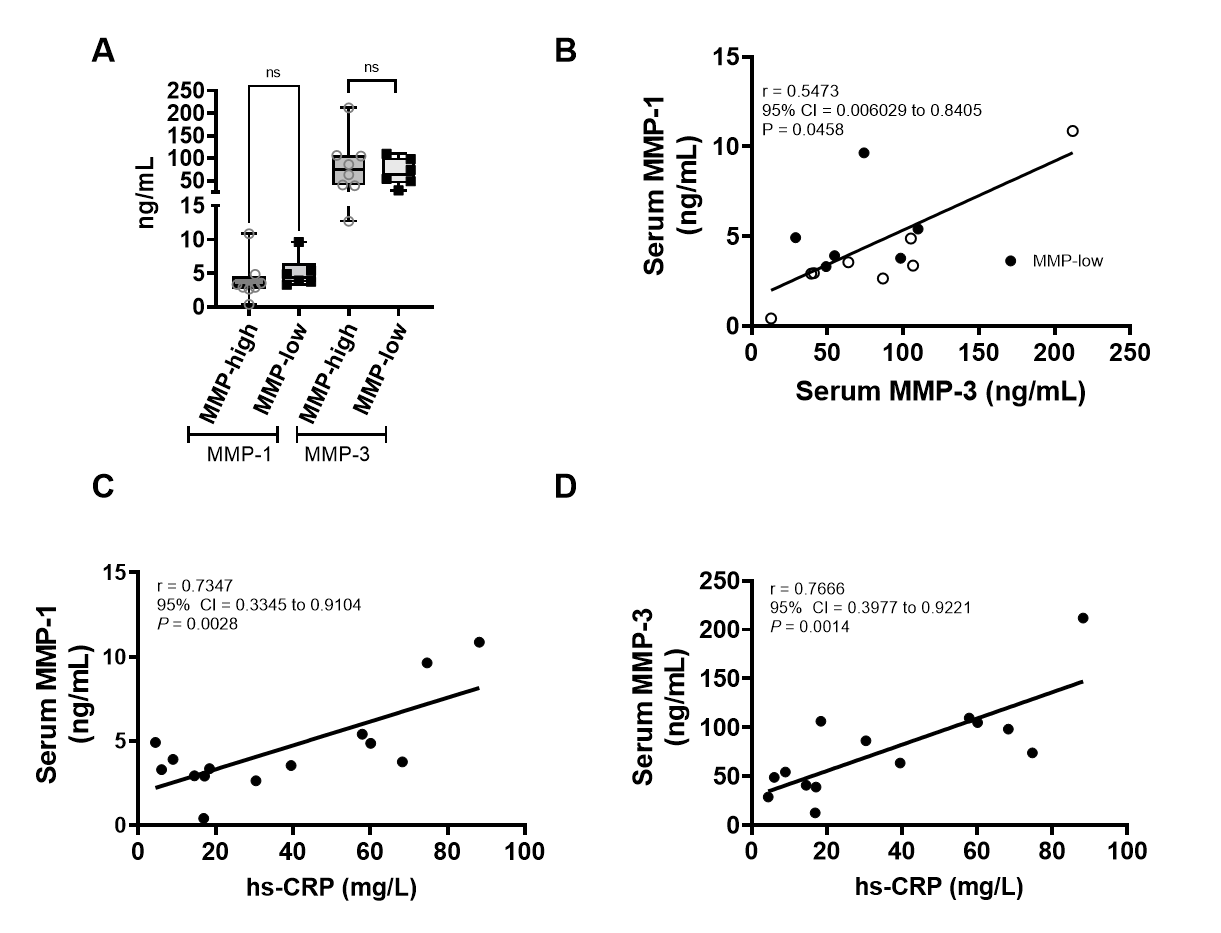


**Table S4:** List of genes that are differentially expressed between MMP-high and MMP-low ERA patients. Data was analyzed using independent samples T test and corrected for multiple comparison using Bonferroni correction


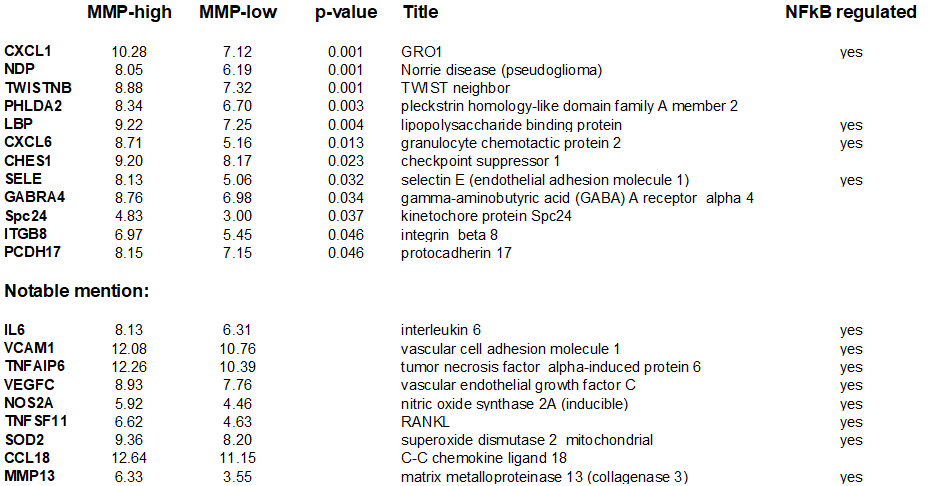


**Table S5**: Table showing baseline clinical features between MMP-high and MMP-low ERA patients. CRP = C-reactive protein, RF = rheumatoid factor, ESR = erythrocyte sedimentation rate, DAS = disease activity score. Data is represented either as mean (standard deviation, SD), n (%) or median (range), as required. Analyzed using either Mann-Whitney test or ^$^Chi-square test.
